# Domestication of a magic therapeutical wine glass fungus (Podoscypha petalodes) from Pakistan

**DOI:** 10.1016/j.heliyon.2023.e16146

**Published:** 2023-05-24

**Authors:** Abdul Rehman Niazi, Aneeqa Ghafoor

**Affiliations:** Institute of Botany, University of the Punjab, Lahore, 54590, Pakistan

**Keywords:** *Podoscypha petalodes*, Culturability, Spawn, Cultivation, Pilot study

## Abstract

The *Podoscypha* is a recognized therapeutically significant genus of mushrooms. A *Podoscypha* species under a *Morus* sp. Was found from the largest man-made forest Changa-Manga, Kasur during the exploration of fungal flora of Pakistan. Detailed morpho-anatomical and phylogenetic analysis identified it as *P. petalodes*, a part of common native flora of many regions of the world like Pakistan. The culturabilty and cultivation potential of this fungus was assessed for the first time using different media and substrates. Maximum cultural growth was observed on the Compost Extract Agar (CEA) medium at 28 °C. Mycelium of cultured strains on CEA medium was used for the spawn production on wheat, sorghum and barley grains. Cultivation potential in the form of spawn running period, harvesting time duration and yield was investigated on variety of substrates. A mixed substrate of sawdust and tea waste at 28 °C showed the optimum yield. Tea-waste was used as the casing material in all substrates and proved very effective. These results depicted that *Podoscypha petalodes* possesses the cultivation potential. Its cultivation on large scale can solve the major health concerns of the growing population. It would provide the people easy accessibility of economical, effective and natural medicine throughout the year that restrict in case of only natural production at specific time of the year.

## Highlights

• Podoscypha petalodes is a magic therapeutic mushroom.
• Podoscyphic acid produced by this fungus effective for viral diseases.
• First time assessed for its culturability and cultivation potential.
• A Pilot Project leads to the commercial production.
• Tea waste + Sawdust proved to be efficient nutritional substrate for its successful growth/cultivation at 28 °C.

## Graphical abstract

**Figure:**
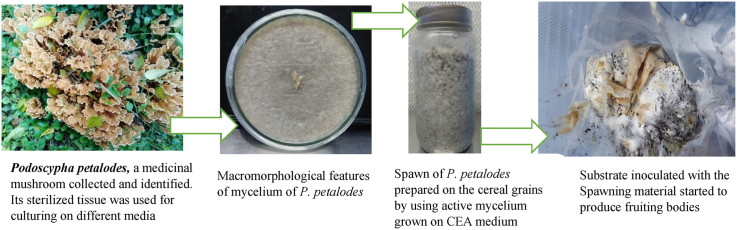

## 1 Introduction

Fungi remained a part of human life since thousands of years with different life strategies. They inhabit soil, wood, dead organic matter or sometimes grow as parasites. These may be macro or micromycetes, gilled or porous, hard or fleshy, stipitate or non-stipitate etc. *Podoscypha* Pat. Is a stipitate stereoid fungal genus belongs to the group Basidiomycetes, order Polyporales and family Meruliaceae [1]. About 49 species of this genus have been reported so far throughout the world [[2], [3], [4], [5], [6], [7], [8], [9], [10], [11], [12]]. The name stipitate stereoid refers to these fungi's resemblance to species of the genus *Stereum* Pers., which have a similar tough consistency and a smooth hymenophore but effused-reflexed instead of stipitate basidiocarps. The life strategies of the stipitate stereoid fungi are poorly known [1] and Reid [5] assumed that all were saprotrophic because many of them were found on deadwood. *Podoscypha petalodes* is also a stipitate stereoid and saprotrophic fungus whose life cycle is not clearly determined. It is a valuable fungus, produces a rosette shaped fruiting body on the basis of which it is commonly called as wine glass fungus [13]. It possesses significant antioxidant potential in the form of flavonoids and phenolic contents [14]. Podoscyphic acid, a fatty acid extracted from the mycelia of *Podoscypha petalodes* is known as recognized antiviral agent. It is highly effective against the leukemia disease and inhibit the Moloney murine leukemia virus reverse transcriptase [15]. Secondary metabolites derived from the *Podoscypha* spp. Also exhibit the antiparasitic activity (*Anti*-Schistosoma, *Anti*-Haemonchus, Anti-Plasmodium, Anti-Leishmania, *Anti*-Trypanosoma) [16,17]. In addition to medicinal values, *Podoscypha* spp. Were also found to play a significant role in the degradation of recalcitrant Polycyclic Aromatic Hydrocarbon compounds like Phenanthrene (PHE) and Pyrene (PYR) in both *in-vitro* and *in-vivo* conditions [18].

*Podoscypha petalodes* possesses both medicinal and biotechnological applications but its culturability and cultivation potential was never assessed before to get maximum advantage from this significant fungal species. The aim of this research work was to determine the mycelial characteristics and growth potential of *Podoscypha petalodes* on the variety of media/substrates and temperatures and optimize its growth requirements. This paper is the first report of successful pilot-scale production of the fruiting bodies of *Podoscypha petalodes* on different lignocellulosic substrates. From these results, it is concluded that *Podoscypha petalodes*, a therapeutic wine glass fungus possesses both the culturability and cultivation potential and by its commercial production, a number of pharmaceutical drugs can be prepared continuously, which delayed in case of only its natural finding in the wild at specific time of the year.

## 2 Material and methods

### 2.1 Sampling, systematic characterization, and experimental design

Basidiomata of the *P. petalodes* were collected on 20th July 2021 during monsoon season (July–September) from the largest man-made forest, Changa Manga, Kasur, Pakistan. The Collected (wild) and harvested (cultivated) specimens were characterized macro-microscopically and phylogenetically according to the literature already available [13,19]. Morphological features such as size, shape and surface features of different parts of the basidiomata were recorded from the fresh specimens. Colors were designated according to Munsell [20]. Micro morphological characteristics were observed using a compound light microscope (MX4300H Techno Co., Ltd., Japan) with an oil-immersion lens at a magnification of 100×. Molecular characterization was done by following the Protocol of Gardes & Bruns [21] with little modifications. The ITS-nrDNA region (Internal Transcribed Spacer of the nrDNA) was amplified using the primer pair ITS1F (forward primer) [21] and ITS4 (reverse primer) [22]. Bidirectional sequences of ITS region were assembled by using BioEdit software [23]. Highly similar ITS sequences were retrieved using Basic Local Alignment Search Tool (BLAST) analysis. The Phylogenetic tree was built through molecular evolutionary genetic analysis using MEGA 6 software with default settings [24]. The sequences under OP341818 and OP341819 accession numbers were deposited in the GenBank. Specimen wild *P. petalodes* (LAH20721)*,* and cultivated *P. petalodes* (LAH20721C) was deposited in the Herbarium, Institute of Botany, University of the Punjab, Lahore, Pakistan for ready reference.

All the experiments i.e., identification, evaluation of culturability, spawn production and determination of cultivation potential were carried out in Fungal Biology and Systematics Research lab, Institute of Botany, University of the Punjab, Lahore. The experiments were arranged in a complete randomized design with three replications per treatment.

## 3 Evaluation of culturability of *P. petalodes*

Culturability of *P. petalodes* was assessed according to the method described by Siddiq et al. [25]. Small tissues from the petal like fruiting bodies were taken and surface sterilized with the help of 5% (w/v) sodium hypochlorite solution and sterile distilled water*.* After surface sterilization*,* with the help of sterile tweezer and blade, mushrooms tissues were placed onto five different nutrient agar media i. e, Malt extract agar (2% MEA: agar 20 g, malt extract 20 g dissolved into 1000 mL dH_2_O), Potato dextrose agar (2% PDA: thin potato slices 200 g, glucose 20 g, agar 20 g per 1000 mL dH_2_O), Glucose peptone agar medium (2% GPA: 20 g peptone, 20 g dextrose, 5 g Nacl, 15 g agar dissolved into 1000 mL dH_2_O), Saboraud dextrose agar (2% SDA: 15 g agar, 40 g dextrose, 10 g peptone dissolved into 1000 mL dH_2_O) and Compost extract agar (2% CEA: 20 g agar, 10 g glucose dissolved into 1000 mL wheat straw water based filtrate). Inoculated petri plates were sealed with parafilm and then incubated at different temperatures i.e., 16 °C, 20 °C, 24 °C, 28 °C, and 32 °C. Mycelial growth characteristics (growth rate, density, texture, color) were observed on regular basis for up to 35 days. The diameter of the mycelium extension rate was measured with the help of a transparent ruler regularly at the same time interval. A Completely randomized design was used to determine the culturability potential on five different media at five different temperatures. Joint effect of media and temperatures was also observed. Each effect was determined in triplicates. The mushroom cultures were also deposited in Herbarium, University of the Punjab, Lahore Culture Collection (as LAH#25721 F C(ABCDE).

### 3.1 Spawn production

Methodology described by Pal & Thupa [26] was followed to prepare spawn. Verified seeds/grains viz., Sorghum, Wheat, and Barley were obtained from the agriculture seed bank, Lahore to use as the substrate to determine the spawn production efficiency. For spawn preparation, grains were washed and soaked in distilled autoclaved water overnight (12 h), boiled for half an hour and excess water was removed by spreading them on blotting paper. Three quarters of each 1 kg filter jars was filled with these grains supplemented with gypsum (2 g) + lime (1 g) and then autoclaved. Spawn was prepared by inoculating mycelial discs from pure CEA culture on the sterilized grains in laminar air flow cabinet. Inoculated grains were incubated at 28 °C. Effect of grains on production of spawning material was determined in triplicates.

### 3.2 Substrate production

Wheat straw, sawdust and tea waste were used as the raw materials. Dried wheat straw collected from the field area of University of the Punjab, Lahore, sawdust of *Morus* species collected from the furniture shop, while tea-waste collected from the Hostel Canteens of University of the Punjab, Lahore. Tea waste is the left-over residue of tea (water containing tea) after usage and enriched with cellulose, hemicellulose, proteins, lipids, polyphenols as well as many minerals [27]. Six types of substrates were prepared. Three of pure types i.e., wheat straw, sawdust and tea-waste while three were of mixed type i.e., sawdust and wheat straw, tea waste and saw dust and of tea waste and wheat straw. For substrate production (pure and mixed types), raw materials were sprinkled with water and made pile of them, 65% moisture was maintained during the substrate production process of ten days. Piles were turned every second day, chicken manure and urea (25 g/kg) were added as supplements for carbon and nitrogen source on the second and last turning while gypsum (15 g/kg) was added and thoroughly mixed before the pasteurization process. When substrates were prepared, they were filled in polypropylene bags and autoclaved for 3–4 h at 121 °C with 15 psi for sterilization purpose. Polypropylene bags of 20 × 15 cm were used and 700 g of the substrates were filled in each bag.

### 3.3 Spawning

Sterilized substrates filled bags on cooling were inoculated with the spawn prepared on sorghum grains at the rate of (wet wt./wet wt.). The mouth of the bags was loosely tied with the rubber bands and incubated at different temperatures. A Completely randomized design was used for spawning on six different substrates at five different temperatures. Experiment was performed in triplicates.

### 3.4 Spawn running

Spawn running on the different substrates at different temperatures was observed. During this process, relative humidity of 70% was maintained by humidifier and ventilation fan. When the spawn running was almost accomplished, 1 cm thick casing layer of sterilized tea-waste (tea containing water) was uniformly made (manually) over the spawned compost to avoid dehydration. After pinhead emergence, bags were transferred to the cropping room at 85% relative humidity maintained through continuous ventilation.

### 3.5 Yield

Yield (Fresh weight basis) of different types of substrates up to three flushes was observed as per 700 g of the substrate bags.

### 3.6 Statistical analysis

Completely randomized design was used to determine the different parameters i.e., culturability, spawn production and cultivation potential. All the treatments were evaluated in triplicates and two-way analysis of variance was applied to determine the significant differences between different treatments. SPSS software package was used for the statistical analysis. Data is also expressed as mean value ± S.E.

## 4 Results and discussion

### 4.1 Morpho-anatomical analysis

#### 4.1.1 *Podoscypha petalodes* (berk.) boidin, revue mycol., paris 24: 230 (1959)

**Fig. 1:**
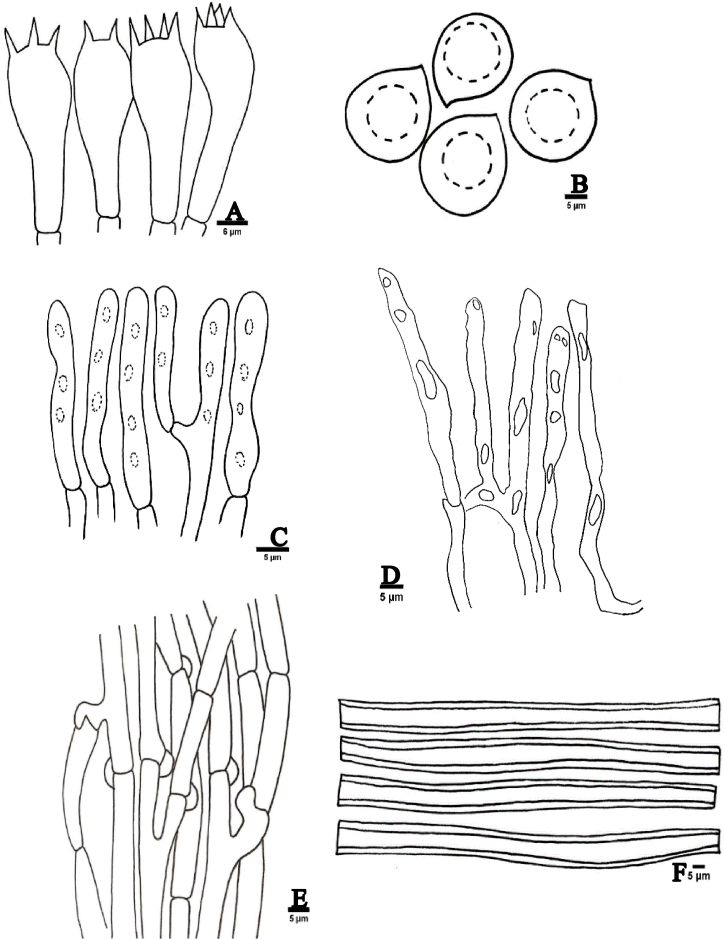
Anatomical features of *Podoscypha petalodes*. A; Basidia, B; Basidiospores, C; Pileocystidia, D; Gloeocystidia, E; Generative hyphae, F; Skeletal hyphae.

Fig. 1(A-F), Fig. 3(A), Fig. 5(A).

**Fig. 2:**
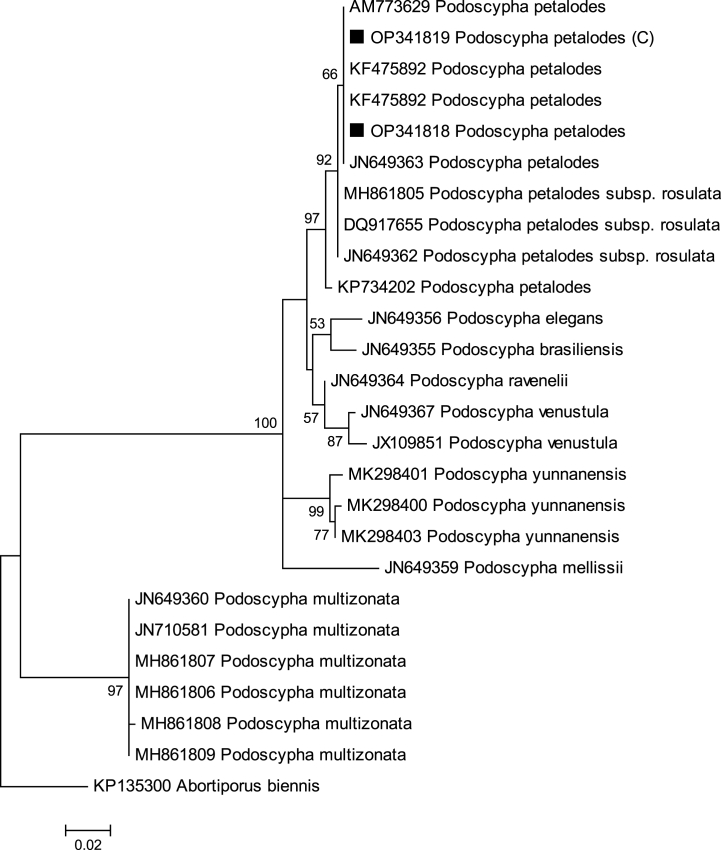
Phylogenetic relationship of *P. petalodes* based on a Maximum Likelihood analysis of the ITS region. The tree was rooted using *Abortiporus biennis*.

**Fig. 3:**
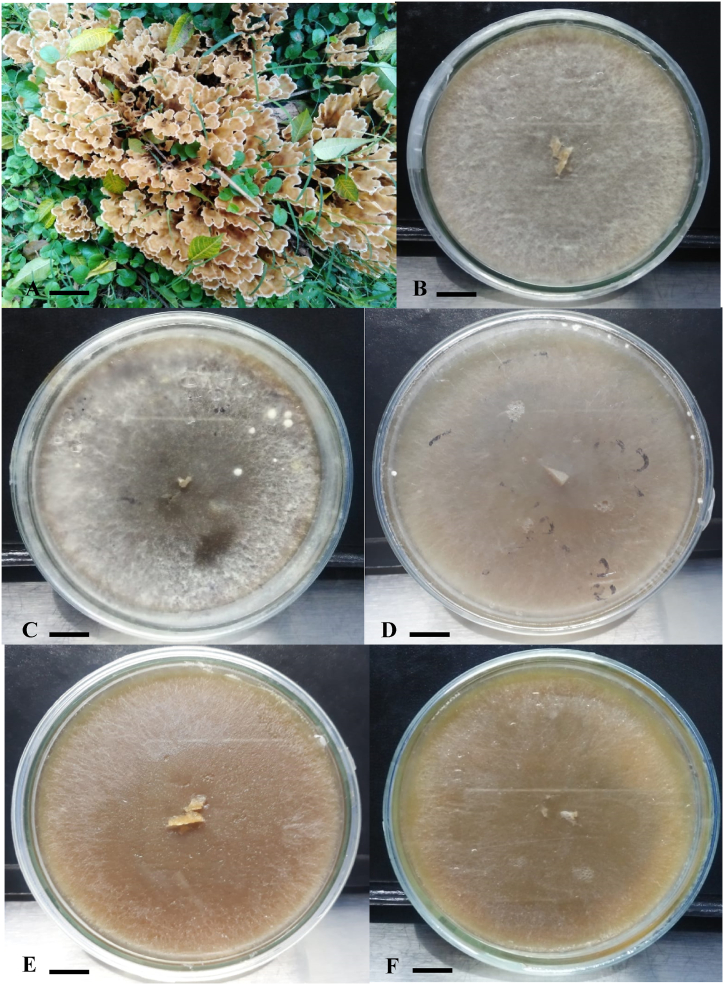
A–F: A; Basidiocarp of *P. petalodes*; B–F: Cultures on different nutrient agar media at 28 °C after 15 days of inoculation; B: on CEA; C: on PDA; D: on GPA; E; on MEA; F: on SDA. Scale bar: A; 2 cm, B–F: 1 cm.

**Fig. 4:**
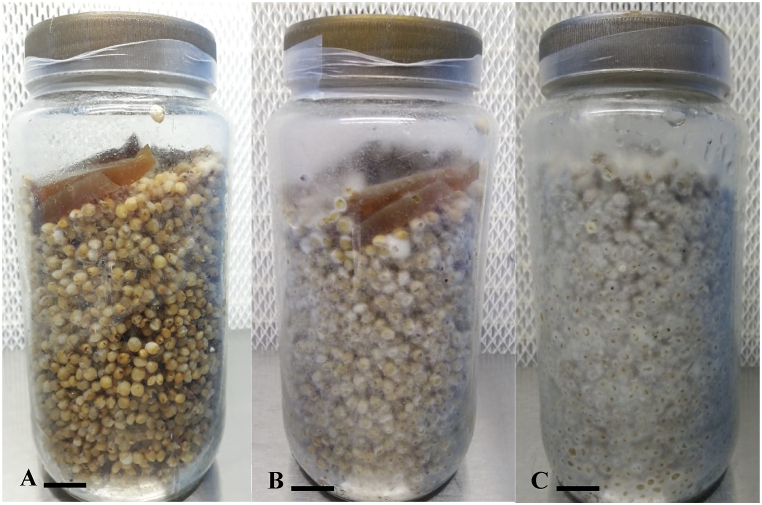
(A, B, C): Complete Spawn production phases of *Podoscypha petalodes* on sorghum grains at 28 °C. A; on 7th day of Inoculation, B; on 13th day, C; on 19th day. Scale bar: A–C: 1 cm.

**Fig. 5:**
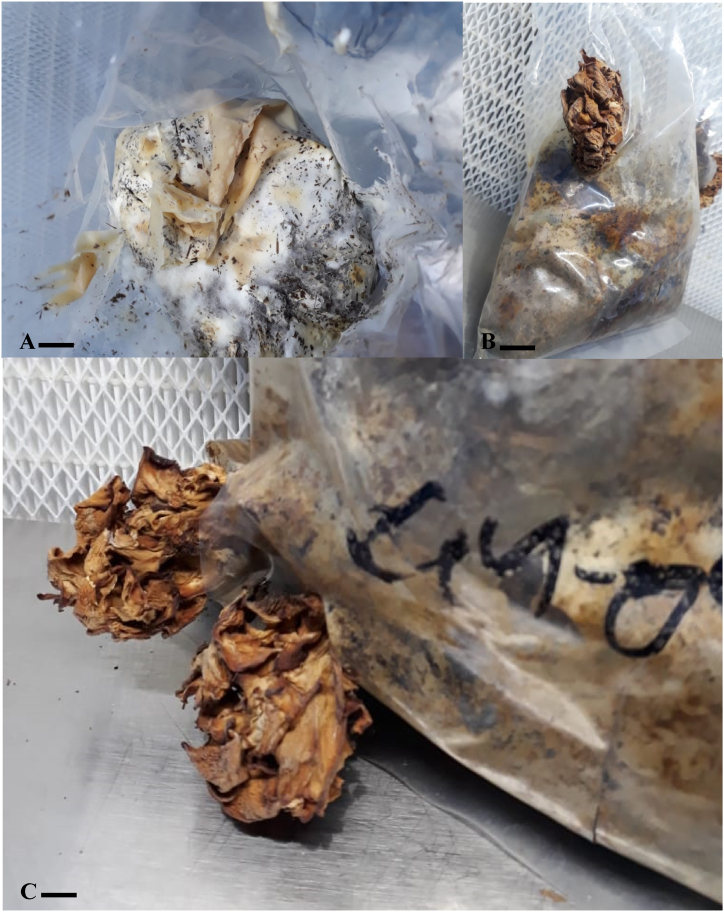
(A, B, C): Different no. Of flushes appeared on the mixture of sawdust and tea-waste substrate. A, first flush on sawdust + teawaste substrate after 10th day of Pinhead formation, B; 2nd flush on 12th day after harvesting of first flush, C; 3rd flush on 16th day after harvesting of 2nd flush. Scale bar; A–C: 2 cm.

**Basidiomata;** spatulous to wine glass shaped, 3.5–7 cm × 1.6–3 cm, light brown to orange brown (8/4 Hue 10 YR) in color with concentric zones, context thin and creamy. Present in Gregarious form, Margin smooth and wavy, 1–1.9 cm long stipe. **Hyphal system dimitic**; Generative hyphae 3.01–4.89 μm, thin-walled, frequently branched, and clamped. Skeletal hyphae 3.45–5.49 μm, unbranched, thick-walled. **Basidiospores**; (3.7–) 3.9–4.85 (–4.96) × (3.2–) 3.5–4.56 (–5.3) μm, avl × avw = 4.54 × 4.23, ovoid to broadly ellipsoid, guttulate, thin-walled. **Basidia**; 19.4–24.79 × 4.75–6.8 μm, narrowly clavate to clavate, 2–4 spored. **Pileocystidia**; 18.67–25.5 × 3.9–6.3 μm, clavate, clamped from the base. **Gloeocystidia**; abundantly present, highly elongated, 23.53–45.82 × 4.86–7.1 μm, thin-walled, narrowly cylindrical to clavate, clamped.

It was the first detailed description of *P. petalodes* on both morpho-anatomical and molecular basis from Pakistan. Previously, Ahmad et al. [28] had reported *P. petalodes* based only on morphological characters.

### 4.2 Molecular and phylogenetic analysis

The aligned data set for phylogenetic analysis of *P. petalodes* (wild and cultivated (C)) include 735 characters including gaps, of which 459 were conserved, 276 were variable sites, 145 were parsimony-informative sites and 74 were singleton sites. In the inferred phylogenetic tree, two in group clades and one outgroup clade were formed (Fig. 2). *Abortiporus biennis* KP135300 was chosen as outgroup. The dataset consisted additionally of 24 sequences comprising 23 in-group taxa of selected *Podoscypha* species and one outgroup taxa.

### 4.3 Screening the most effective culture medium and temperature optimization

Mycelium extension rate and density always rely on the suitable culture medium utilized for culturing in the laboratory [29]. Mycelial characteristics of *P. petalodes* were assessed on different nutrient agar media at different temperatures. Mycelium extension pattern was unique and irregular. Mycelium growth started with light fibrillar hyphae spread throughout the plate. With the passage of time, mycelium started to condense from growing tips towards back along with semi-cottony texture (Fig. 3 (A-F)). Same mycelium growth pattern and texture were observed on all the media tested for its culturability potential. Welden & Bennett [30] observed the plumose type appearance of cultural hyphae of the *Podoscypha multizonata* and *P. ravenelii* on MEA medium*.*

Amongst the different media utilized for the culturing of *P. petalodes,* most rapid growth rate of mycelium (mm/day) was observed on the CEA media (11.96 ± 0.03) at 28 °C followed by PDA (9.96 ± 0.03), GPA (9.56 ± 0.03) MEA (6.96 ± 0.63), and SDA (5.96 ± 0.03). Growth is relatively slow on SDA which might be due to nutritious requirement of the *P. petalodes* mycelium fulfilled quickly by the other media as compared to the SDA medium. Niazi & Ghafoor [31] also revealed the CEA medium as the supportive medium for the culture growth of *Pleurotus floridanus*. Our results were similar with Shim et al. [32] in which they found PDA medium as the suitable medium for the cultural characteristics of *Cystoderma amianthinum.* These results were in agreement with work of Roy & Krishnappa [33]. They used PDA, SDA, MEA and czapek dextrose agar media (CZA) for mycelial growth of *Macrocybe gigantea* and found PDA as the best medium for maximum mycelial growth of *M. gigantea*. The current findings were also similar with the work of Jo et al. [34] in which they screened the PDA and MEA media as the optimum culture media for the *Coriolus versicolor.*

It is recognized that temperature plays a vital role in the growth of fungal mycelium in natural or artificial conditions. Different basidiomycetous species grow in a varied temperature, particularly between 20 and 30 °C [[35], [36], [37], [38], [39]]. The temperature, 28 °C proved to be the optimum temperature for the mycelial growth of *P. petalodes* and growth remains suppressed at 32 °C. The findings were also concurrent with the response of the various tropical mushrooms like *Lentinus squarrosulus* [40], *Calocybe indica* [41,42], *Collybia reinakeana* [43], *Schizophyllum commune* [44], *Volvariella volvacea* [45] and *Lentinus tigrinus* [46]. Lai et al. [47] observed the same pattern of mycelial growth in *Lignosus rhinoceros*. Vigorous mycelial growth was observed with rise in temperature but remains stunted after 30 °C. The current findings also corroborate with those of Shim et al. [48] in which mycelial growth of *Macrolepiota procera* was suppressed at 35 °C. After these findings, it is concluded that all media with varied growth rate proved to be supportive for the mycelial growth of *P. petalodes* which showed its facultative saprotrophic nutrition habit. Mycelium extension rate (mm/day) on different media at different temperatures was significantly different (Table 1).

**Table 1:** Mycelium extension rate (mm/day) of *P. petalodes* at different temperatures on different media.

Types of media	Mycelium extension rate (mm/day)					
	Temperatures					
	16 °C	20 °C	24 °C	28 °C	32 °C	***P*-value**
CEA	4.96 ± 0.03	7.40 ± 0.05	9.33 ± 0.03	11.96 ± 0.03	10.85 ± 0.03	<0.001
PDA	5.96 ± 0.03	7.93 ± 0.03	8.96 ± 0.03	9.96 ± 0.03	8.95 ± 0.04	<0.001
MEA	3.95 ± 0.04	6.96 ± 0.03	7.43 ± 0.05	8.26 ± 0.63	6.96 ± 0.03	<0.001
SDA	1.96 ± 0.03	2.96 ± 0.03	3.4 ± 0.05	5.96 ± 0.03	3.96 ± 0.03	<0.001
GPA	4.93 ± 0.03	6.96 ± 0.03	8.86 ± 0.03	9.56 ± 0.03	8.93 ± 0.03	<0.001
***P*-value**	<0.001	<0.001	<0.001	<0.001	<0.001	

Values given are mean ± Standard error. Media type and temperature have significant impact over Mycelium growth rate (p < 0.001). Moreover, the joint effect of media and temperature has also a significant impact over Mycelium extension rate (p < 0.001).

CEA, Compost Extract Agar; PDA, Potato dextrose agar; MEA, Malt Extract Agar; SDA, Saboraud Dextrose Agar; GPA Glucose Peptone Agar.

### 4.4 Spawn production efficiency of *P. petalodes on* sorghum, wheat and barley grains

The grain spawn is the initiator for the mass/bulk production of mushroom. It promotes the quick colonization of the mycelium that is essential for successful mushroom fruiting [49]. Colonization rate of the active mycelium (cultured on the CEA medium) on cereal grains (sorghum, wheat and barley grain) was checked at 28 °C. Mycelium colonized more quickly on sorghum grains at 28 °C as compared with wheat grains. Sorghum grains showed higher efficiency in terms of minimum days required for the production of spawning material followed by the wheat and barley grains. Sorghum grains are commonly used for spawn production of many macrofungi, as it is easily accessible, cheap, and because of its ability to soak optimal amount of water [50]. The luxuriant mycelial growth on sorghum grains could also be due to their nutrient composition. According to Leder [51], 100 g sorghum seeds comprise 10.9 g protein, 2.3 g crude fiber, 3.2 g fat, 1 g ash, 329 kcal of energy, 27 mg calcium, 4.3 mg iron, 3.83 mg niacin, 0.3 mg thiamin, and 0.138 mg riboflavin. Same mycelial colonization pattern was observed during spawn production as observed in pure culture media of *P. petalodes.* Following developmental stages were observed during spawn production. Firstly, light mycelial growth covered the whole grains in bottle (on 13th day on sorghum grains) then gradually becomes thick with the passage of time (Fig. 4 (A,B,C). Our findings related to that of Rizal et al. [52], Dulay et al. [53], Devi & Sumbali [54], as they found maximum mycelial colonization of *Macrolepiota detersa*, *Trametes versicolor,* and *M. gigantea,* on sorghum grains in minimum no. Of days. Stanley et al. [55] investigated the spawn production efficiency of *Pleurotus tuber-regium* and *Pleurotus pulmonarius.* They prepared spawn on different cereal grains viz., Wheat, yellow maize, Guinea Corn, Millet, Red Sorghum and White Maize. They found red sorghum grains as best spawn production medium. Sorghum grains was also found to be the good spawning material for *Lentinus sajor-caju, Agaricus blazei, Auricularia polytricha and Agrocybe aegerita* [[56], [57], [58], [59]]. Table 2 showed that the days required to complete spawn production of *P. petalodes* on sorghum, wheat and barley grains at 30 °C significantly differed at (p < 0.001).

**Table 2:** Efficiency of wheat, sorghum and barley grains for spawn production of *P. petalodes*.

Types of grains	Days required to complete spawn production at 28 °C
Sorghum	19.5 ± 0.05
Wheat	24 ± 0.03
Barley	27 ± 0.03

The results reported were run in triplicates and stated as Mean ± Standard error.

### 4.5 Determination of efficient lignocellulosic substrates for fruiting of *P. petalodes*

The success of a newly cultivated strain depends on both economical and biological factors [60]. Temperature is one of the most important biological factors for the successful fruiting of any mushroom or the conversion of the dikaryotic mycelium into the fruiting body. Mata et al. [61] reported that different factors like, temperature, light and humidity of the incubation room influence the spawn running time of mushrooms. Spawn prepared on the sorghum grains was used to determine the spawn running time on different substrates at various temperatures viz., 16 °C, 20 °C, 24 °C, 28 °C, and 32 °C. At 28 °C, Spawn running completed with less incubation time on all substrates. However, tea waste + sawdust substrate (22.96 ± 0.03) proved to be the best. (Table 3). Further, cultivation potential (fruiting potential, harvesting time, yield and no. Of flushes) was investigated at 28 °C on six types of substrates. These were the pure wheat straw, mixture of sawdust and wheat straw, pure sawdust, mixture of tea waste and saw dust, pure tea-waste, and mixture of tea waste and wheat straw. Mixture of tea waste and saw dust proved the most efficient substrate medium for the fruiting of *P. petalodes* as maximum yield was obtained from this substrate (296.88 ± 0.03 g) followed by pure tea-waste (266.85 ± 0.03 g). Minimum period (10 days) from pinhead to the first mature flush was also found on the sawdust + teawaste substrate. While harvesting of first flush on all other substrates required more than 15 days (Table 4 and Fig. 5 (A, B, C)). As far as the efficiency of substrates were concerned, our results were in accord to the findings of Peksen & Yakupoglu [62] and Dulay et al. [63] as they found the highest yield and biological efficiency of *Ganoderma lucidum* and *Lentinus* species respectively from the sawdust-based substrates. Baktemur et al. [64] also investigated that highest yield of *P. ostreatus* obtained from the substrate containing tea waste. However, our results were different from the Rupasinghe & Nandasena [65] in which they experimented the poor yield of oyster mushrooms from the substrate of equal mixture of sawdust and tea waste and pure tea waste while the highest yield obtained from the sawdust substrate. In this experiment, tea-waste was used as the casing material that was also proved a significant and economical casing material. Peyvast et al. [66] observed the mixture of tea waste and traditional peat as the best casing material for the highest yield of *Agaricus bisporus*. Gulser & Pekşen [67] also not found any significant difference between the *A. bisporus* yields of tea waste + peat and peat casing materials at the end of harvesting.

**Table 3:** Days required to complete Spawn Running Period of *P. petalodes* on different substrates at variable temperatures.

Days required to complete Spawn running period					
Types of substrates	Temperatures				
	16 °C	20 °C	24 °C	28 °C	32 °C
Tea waste & saw dust	28.96 ± 0.03	24.93 ± 0.03	26.96 ± 0.03	22.96 ± 0.33	23.99 ± 0.06
Pure tea waste	27.93 ± 0.03	26.93 ± 0.05	26.96 ± 0.06	24.93 ± 0.05	24.93 ± 0.03
Tea waste & wheat straw	29.92 ± 0.03	27.96 ± 0.06	26.96 ± 0.03	24.96 ± 0.03	26.96 ± 0.03
Pure wheat straw	35.93 ± 0.03	33.96 ± 0.03	32.93 ± 0.05	32.96 ± 0.03	32.95 ± 0.03
Pure saw dust	30.96 ± 0.03	29.93 ± 0.05	27.96 ± 0.03	26.96 ± 0.03	27.93 ± 0.03
Saw dust & wheat straw	31.93 ± 0.03	29.91 ± 0.04	25.96 ± 0.06	25.93 ± 0.05	27.96 ± 0.03

Values given are mean ± Standard error. Substrate types and temperature have significant impact over spawn running time (p < 0.001). Moreover, the joint effect of substrates and temperature has also a significant impact over spawn running time (p < 0.001).

**Table 4:** Yield of *P. petalodes* (fresh weight basis) obtained from different types of substrates.

Types of Substrates	Yield (g)			
	1st flush yield	2nd flush yield	3rd flush yield	Total yield
Tea waste and Sawdust	110.96 ± 0.03	89.96 ± 0.03	95.96 ± 0.03	296.88 ± 0.03
Pure tea waste	99.96 ± 0.03	85.96 ± 0.03	80.93 ± 0.03	266.85 ± 0.03
Tea waste and wheat straw	74.96 ± 0.03	70.93 ± 0.03	Not appeared	145.89 ± 0.03
Pure wheat straw	61.96 ± 0.03	58.94 ± 0.04	Not appeared	120.9 ± 0.03
Pure saw dust	75.4 ± 0.05	73.93 ± 0.06	Not appeared	149.33 ± 0.05
Saw dust and wheat straw	67.93 ± 0.03	63.93 ± 0.03	Not appeared	131.86 ± 0.04

Values given are mean ± Standard error. Substrate types have significant impact over the total yield (p < 0.001). 700 g of substrate was used in each experiment.

## 5 Conclusion

The present study demonstrated for the first time the pilot scale production of *Podoscypha petalodes*. In conclusion, CEA medium at 28 °C was found as the most supportive conditions for mycelial growth of *P. petalodes* in terms of both density and growth rate. For the production of spawning material, sorghum grains was found to be the most effective medium. The teawaste + sawdust at 28 °C proved to be the most reliable substrate for the spawn running or vegetative growth and reproductive growth. Minimum period of flush harvesting i.e., on 10th day from pinhead emergence, thick flesh content, maximum yield i.e., 296.88 g/700 g on fresh weight basis was also observed on teawaste + sawdust substrate with 85% humidity and proper ventilation*.* Hence these findings showed that this noteworthy fungus can grow on many economical media and substrates. However, teawaste + sawdust medium proved to be the best for growth medium and also as the casing material. Nevertheless, different combinations of the substrates should be investigated in more detail to enhance the yield and biological efficiency. Further detailed studies on the optimization of culturing and basidiocarp cultivation are required to kick start its commercial production and can make best possible utilization of its therapeutic potential for the formation of significant pharmaceutical drugs or medicine in future.

## Author contribution statement

Abdulrehman Niazi: Conceived and designed the experiments; Analyzed and interpreted the data; Contributed reagents, materials, analysis tools or data.

Aneeqa Ghafoor: Performed the experiments; Wrote the paper.

## Data availability statement

Data associated with this study has been deposited at Specimens and cultures have been deposited in the public herbarium, University of the Punjab, Lahore, Pakistan (LAH) and cultures have been deposited in the culture collection as LAH#25721 F C(ABCDE).

## Declaration of interest's statement

The authors declare no conflict of interest.

## Additional information

Supplementary content related to this article has been published online at [URL].

## Funding statement

There is not any funding associated with this article.

## Declaration of competing interest

The authors declare that they have no known competing financial interests or personal relationships that could have appeared to influence the work reported in this paper
